# Remote Sensing Evaluation of Total Suspended Solids Dynamic with Markov Model: A Case Study of Inland Reservoir across Administrative Boundary in South China

**DOI:** 10.3390/s20236911

**Published:** 2020-12-03

**Authors:** Jing Zhao, Fujie Zhang, Shuisen Chen, Chongyang Wang, Jinyue Chen, Hui Zhou, Yong Xue

**Affiliations:** 1 Guangdong Open Laboratory of Geospatial Information Technology and Application, Key Lab of Guangdong for Utilization of Remote Sensing and Geographical Information System, Guangdong Engineering Technology Center for Remote Sensing Big Data Application, Guangzhou Institute of Geography, Guangdong Academy of Sciences, Guangzhou 510070, China; b20203080626@cau.edu.cn (J.Z.); wangchongyang@gdas.ac.cn (C.W.); chenjinyue18@mails.ucas.ac.cn (J.C.); zhouhui@imapcloud.cn (H.Z.); 2 Faculty of Agriculture and Food, Kunming University of Science and Technology, Kunming 650500, China; 20030031@kust.edu.cn; 3 College of Information and Electrical Engineering, China Agricultural University, Beijing 100083, China; 4 College of Engineering and Technology, University of Derby, Derby DE22 1GB, UK; Y.Xue@derby.ac.uk

**Keywords:** total suspended solids, progress degree, Markov model, remote sensing, river chief system

## Abstract

Accurate and quantitative assessment of the impact of natural environmental changes and human activities on total suspended solids (TSS) concentration is one of the important components of water environment protection. Due to the limits of traditional cross-sectional point monitoring, a novel water quality evaluation method based on the Markov model and remote sensing retrieval is proposed to realize the innovation of large-scale spatial monitoring across administrative boundaries. Additionally, to explore the spatiotemporal characteristics and driving factors of TSS, a new three-band remote sensing model of TSS was built by regression analysis for the inland reservoir using the synchronous field spectral data, water quality samples and remote sensing data in the trans-provincial Hedi Reservoir in the Guangdong and Guangxi Provinces of South China. The results show that: (1) The three-band model based on the OLI sensor explained about 82% of the TSS concentration variation ($R^{2}=0.81,\;N=34,\;\;p\;\mathrm{value}<0.01$) with an acceptable validation accuracy (RMSE=6.24 mg/L,MRE=18.02%, N=15), which is basically the first model of its kind available in South China. (2) The TSS concentration has spatial distribution characteristics of high upstream and low downstream, where the average TSS at 31.54 mg/L in the upstream are 2.5 times those of the downstream (12.55 mg/L). (3) Different seasons and rainfall are important factors affecting the TSS in the upstream cross-border area, the TSS in the dry season are higher with average TSS of 33.66 mg/L and TSS are negatively correlated with rainfall from upstream mankind activity. Generally, TSS are higher in rainy seasons than those in dry seasons. However, the result shows that TSS are negatively correlated with rainfall, which means human activities have higher impacts on water quality than climate change. (4) The Markov dynamic evaluation results show that the water quality improvement in the upstream Shijiao Town is the most obvious, especially in 2018, the improvement in the water quality level crossed three levels and the TSS were the lowest. This study provided a technical method for remote sensing dynamic monitoring of water quality in a large reservoir, which is of great significance for remediation of the water environment and the effective evaluation of the river and lake chief system in China.

## 1. Introduction

Total suspended solids (TSS) are essential carriers of organic matter such as nitrogen and phosphorus and their movement and migration play a significant role in the process of global material cycling and change [1]. By influencing the distribution of scattered light in the water body, TSS directly control the primary productivity of the water body [2], which in turn affects the transparency and oxygen content of the water body, and exert a decisive part in the aquatic ecological environment. Reservoirs are one of the most important sources of drinking water for human beings. They are rich in biodiversity and play a decisive role in improving and regulating the surrounding ecological environment. At the same time, the spatial and temporal heterogeneity of TSS and the process of resuspension and flocculation also directly affect the morphological dynamics of the reservoir [3,4]. As a result, TSS are essential for understanding the progress of river sediment transport and water quality variation. The reservoir gradually accumulates suspended matter at different water levels, which reduces the water storage capacity of the reservoir and ultimately reduces the effective storage capacity [5]. Therefore, it is particularly important for real-time monitoring and management of the aquatic environment in the reservoir area to reveal the dynamic of TSS. Since the release of the guidance for the river chief system by the General Office of the State Council of China in 2016, the research of TSS in inland water including lakes and reservoirs has been paid more lasting attention, and the associated research has been conducted by a variety of scholars, governments and social communities [6,7,8], such as the Río Tercero reservoir (Córdoba, Argentina) [6], the Mekong and Bassac Rivers [9,10], the Amazon River [11], Taihu Lake [8,12], the Yangtze River [13,14,15] and the Pearl River [16,17,18,19].

There are many methods used for monitoring the TSS concentration and the spatial and temporal variation assessment, such as hydrological fixed-site monitoring, in situ cruising investigation, physical models, numerical simulation, remote sensing and so on. Fortunately, as a scientific and rapid investigation tool, remote sensing breaks through the time-consuming, tedious and expensive limitations of traditional monitoring technologies, and it has been considered as a superior method with the advantages of wide coverage, periodic revisits and long series of data collection. With the continuous improvement in remote sensing data in spectral, spatial and temporal resolution, the use of different types of remote sensing data for TSS monitoring and evaluation is increasingly accepted and recognized. There are numerous remote sensing data for assessment of TSS including the series of Land Observation Satellite (Landsat) [18,20,21,22], the Moderate Resolution Imaging Spectroradiometer (MODIS) [11,12,23,24,25], the Geostationary Ocean Color Imager (GOCI) [8,26,27], Environment and Disaster Monitoring and Forecasting Small Satellite Constellation (HJ) [28], Medium Resolution Imaging Spectrometer (MERIS) [9] and so on. Among them, due to the high quality, high spatial resolution and inheritance, Landsat has become the most widely used remote sensing sensor in spatiotemporal dynamic analysis. 

The commonly used TSS retrieval models are divided into empirical models and analytical models. As far as the empirical model is concerned, it does not need to undergo complicated derivation and parameter initialization, and its form is simple with sufficient accuracy, which is highly efficient to use and will continue to be used for a long time in the future. Although many studies use empirical models for TSS estimation from different sensors, there are significant differences in the band selection of single- or multiple-band models [12,18,24,29,30]. First, the sensitivity of satellite sensors to different concentrations of TSS is different. The study by [13] showed that the red band reflectance is positively correlated with TSS, but when TSS reach a certain level, the reflectance will tend to converge or remain unchanged. Besides, the difference in optical characteristics of different component water bodies makes the TSS-sensitive bands different among open ocean, coastal and inland water bodies. Compared to open ocean and estuary coastal waters, inland waters are optically more heterogeneous and complex [31]. The reason is probably because the different phytoplankton types in estuaries and coasts are different from those in Hedi Reservoir, such as diatoms or cyanobacteria. The different types of phytoplankton produce different spectral properties in the water. Therefore, it is necessary to develop a reliable model based on the specific spectral properties of water constituents for retrieving the temporal and spatial distribution of TSS by remote sensing.

No doubt analyzing the spatial-temporal trends of TSS is important for studying the evolution process and effect of lake and reservoir water environment systems. Some related research works have made important achievements in their respective fields, such as Taihu Lake [8,12], Dongting Lake [32,33], reservoirs [5,22] and so on. Zheng et al. [32] conducted remote sensing assessments of environmental changes and human economic activities on the variation patterns of the concentration of TSS, revealing their significant inter-annual and spatial variations. Unfortunately, although studies have been able to use large amounts of data to analyze the changes in TSS [15,19,31], few studies can be combined with local policy strategies to comprehensively analyze TSS changes [7,34]. Moreover, fewer studies focus on cross-border areas where the water quality change is more complicated from upstream runoff. To achieve the goal of the development of Green Water and Green Hills that China’s government put forward, it has become key to systematically study the long time series dynamic changes in the water quality of reservoirs and understand the impact mechanism for water environment management and pollution prevention by different river chiefs.

A new method of combining the Markov process and remote sensing technology is used to reveal the spatiotemporal heterogeneity of TSS, which is based on the advantage of a Markov model that reflects the stochastic dynamic trends of events during a certain period. Large-scale and long-term monitoring has replaced the traditional cross-section in situ sampling [35], which makes the monitoring process faster and more concise and can more intuitively reflect the dynamic and continuous change process of the TSS concentrations. In the study, based on the field spectra and remote sensing experiments carried out in the cross-border watershed Hedi Reservoir of western Guangdong in South China, we built the TSS three-band retrieval model, analyzed the spatial and temporal distribution characteristics of TSS concentration and then explored the driving factors. The dynamic changes in TSS in the Hedi Reservoir were analyzed from 2014 to 2018 based on the Markov model. The quantitative assessments of TSS variations and their driving force factors were produced for the effect analysis of the river and lake chief system in China.

## 2. Materials and Methods

### 2.1. Study Area

**Figure 1:**
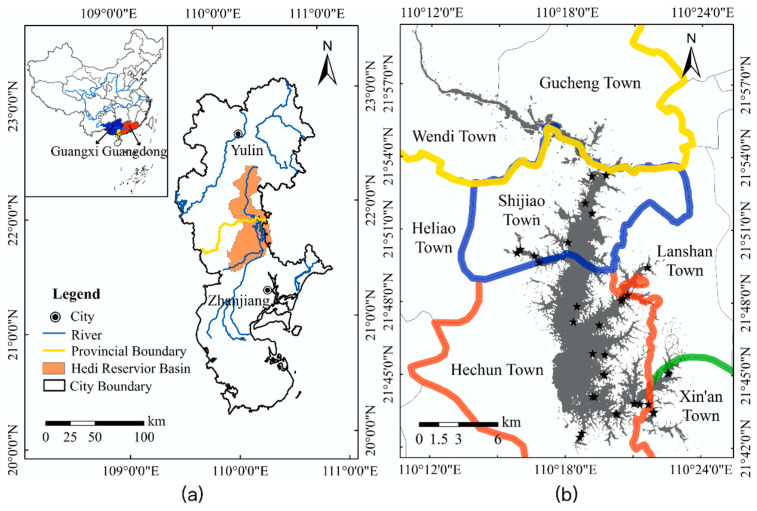
(**a**) Location of the study area (Hedi Reservoir) and the location of the Hedi Reservoir Basin across Zhanjiang, Guangdong Province, and Yulin, Guangxi Province, in China, and (**b**) location of in situ data labeled by black stars and the distribution of sampling stations where different color lines represent different boundaries of towns.

Crossing the borders of Guangdong and Guangxi Provinces, Hedi Reservoir is located in Hechun Town, Lianjiang City (county level), Zhanjiang City, in Guangdong Province at latitudes of 21°22′–22°22′ N and longitudes of 109°54′–110°26′ E (Figure 1). As a typical trans-provincial basin (Guangdong–Guangxi), the Hedi Reservoir Basin in western Guangdong of China is an important source of drinking water for the two provinces. It undertakes the mission of supporting the sustainable development of the regional economy and maintaining the stability of the regional society. It is a veritable “life water” and “economic water” which has important strategic significance for the ecological protection of the basin. The islands in the reservoir are dotted, and the shoreline twists and turns, forming a large number of backwaters and forks. Its shape is like an olive nucleus. With a total storage capacity of 1.151 billion m^3^ and a catchment area of 1440 km^2^, Hedi Reservoir is a key source of water supply security in western Guangdong and one of the five major drinking water sources in Guangdong Province [36]. The spatial and temporal distribution of TSS in the reservoir is not only closely related to local human activities such as navigational transportation, aquiculture and even illegal-profit business processes [32,37], but also becomes the focus of attention for sustainable development in human society especially in the planning of ecological preservation areas and urban construction [17]. In recent years, the rapid economic development and the increasing human activities have caused some river sections to be polluted to varying degrees, and the water use in the basin has become increasingly tense. The water pollution problem engendered needs to be solved urgently. Correspondingly, China has also adopted relevant measures to solve the problems of rivers and lakes water quality management and protection. That is, the main leaders of the government at all levels in China as the “river chief” (the first person responsible) are responsible for organizing and leading the management and protection of rivers and lakes in the corresponding regions.

### 2.2. Experimental Data and Remote Sensing Imagery

Several satellite-synchronous remote sensing experiments in Hedi Reservoir were conducted between August and October 2015, and water surface spectra and water samples were collected. A total of 35 water samples were collected in this reservoir in August (N = 12) and October ((N = 23), 2015, including 29 valid samples (Figure 1b). Further, we referred to the measured TSS data from Poyang Lake, China’s largest freshwater lake, to increase the applicability of the model. Poyang Lake’s TSS data used in this paper were obtained by Liu et al. [38] and Huang [39] in June 2017. The data include a total of 36 samples and 19 among them with valid spectral data. Table 1 summarizes the variability of all the collected TSS concentrations from different locations. In total, there are 49 measured TSS data that range from 4 to 66 mg/L. All the data are divided into two parts, which are used for model calibration (34 sites) and validation (15 sites).

#### 2.2.1. Synchronous Field Spectral Data

Based on the above-surface spectra measurement method [40], the ASD Field Spec Pro portable spectrometer was used to collect the water surface spectral information, mainly including the water surface radiance and the total irradiance of the water surface incident. The water surface remote sensing reflectance is calculated as Equation (1):

(1) $$R_{rs}=\frac{L_{w}}{E_{d}(o^{+})}$$

where $R_{rs}$ represents the remote sensing reflectance calculated by Equation (1) (units: ${\mathrm{sr}}^{-1}$), $L_{w}$ is the water surface radiance (units: $w\cdot m^{-2}\cdot {\mathrm{sr}}^{-1}$) and $E_{d}(o^{+})$ is the total irradiance of the water surface incident (units: $w\cdot m^{-2}$); both $L_{w}$ and $E_{d}(o^{+})$ are measured by ASD spectrometer equipment.

#### 2.2.2. Water Quality Data

Water quality was represented by the TSS concentration that was measured by the weighing method according to the Chinese national standard of GB11901-89. The TSS concentrations were produced by dividing the mass of the TSS by the volume of the filtered water sample, among which the quality of suspended matter was obtained by placing the filtered sample in an oven at 40–80 °C for 8 h. It is worth noting that when we calculated the volume of the water sample, the water sample filtered by a GF/F filter with a pore size of 0.47 μm was used.

Following the characteristics of the TSS in the reservoir area and actual needs, and referring to the Chinese Standards for Pollutants in Municipal Wastewater Treatment Plants (GB18918-2002), the concentration of TSS is divided into four levels for analysis as follows (Equation (2)).

(2) $$\mathrm{TSS}(\mathrm{mg}/L)\left\{ \begin{array}{l} Ⅰ\;0\leq \mathrm{TSS}<20\;\\ Ⅱ\;20\leq \mathrm{TSS}<30\\ Ⅲ\;30\leq \mathrm{TSS}<50\\ Ⅳ\;\;\mathrm{TSS}\geq 50 \end{array}\right.$$

#### 2.2.3. Remote Sensing Data 

Due to the cloudy and rainy weather in the study area, it is easy to result in a long-term sequence of single optical remote sensing data being missing. Therefore, this experiment uses data from multi-source satellites for analysis. Multi-source satellite images include Landsat8 OLI and Sentinel-2 data. The Landsat 8 satellite is the latest generation of terrestrial remote sensing satellites. Its sensors have been significantly improved in terms of imaging mode, the band setting, signal-to-noise ratio, etc. In contrast, Sentinel-2 has a higher spatial resolution (10 m) and shorter revisit period (5 days), which can supplement missing data in time. Collected satellite data from 2014 to 2018 (29 scenes) consider the different seasons, including 11 scenes during the dry season (November to April of next year), and 18 scenes during the flood period (May to October of each year) (Table 2).

The processing of remote sensing data is mainly based on ENVI and ArcGIS platforms, including radiation correction, FLAASH atmospheric correction, band fusion and other operations. Its purpose is to eliminate errors caused by atmospheric scattering, absorption and reflection.

### 2.3. Methodology

Combined with the spectral characteristics of the water body in the study area, this study builds and optimizes the TSS retrieval model based on field data and quasi-synchronous satellite imageries. By comparing and verifying different TSS retrieval models, comprehensively considering the accuracy errors and applicability of various models, the optimal TSS retrieval model is obtained. Finally, on the basis of the optimal TSS model, we analyzed the spatial and temporal changes in the TSS concentration using the remote sensing data and evaluated the dynamic trends of multi-time series TSS concentration with the help of the Markov model.

#### 2.3.1. TSS Retrieval Model

Based on different remote sensing data, combined with analysis, semi-analysis/semi-experienced and empirical methods, many experts and scholars [10,14,18,22,41,42,43] have calibrated a large number of remote sensing retrieval models for TSS in water bodies, and have obtained good retrieval accuracy with wide applications. In addition, there are many forms of TSS models, mainly including the single-band model, the band ratio model and the multi-band combination model. The band ratio model can effectively reduce the effect of particle size distribution and the bidirectional reflectance change of variable sediment [23]. The three-band model considers the interference of chlorophyll on the remote sensing reversion of the concentration of TSS [44]. Fully considering the optical complexity of the turbid case II waters and the interaction between the spectral characteristics of different water components (chlorophyll, suspended matter and colored dissolved organic matter) and minimizing the TSS concentration error in the remote sensing estimation, this paper mainly uses the band ratio and three-band TSS models in the forms

(3) $$\mathrm{TSS}\propto \frac{R_{rs}(b_{2})}{R_{rs}(b_{1})}$$

(4) $$\mathrm{TSS}\propto \left[R_{rs}{\left(b_{1}\right)}^{-1}-R_{rs}{\left(b_{2}\right)}^{-1}\right]\times R_{rs}(b_{3})$$

where $R_{rs}(b_{i})$ is the reflectance in spectral band $b_{i}$.

To find the best combination of band reflectances, a Pearson correlation analysis was used to investigate the strength of the association between the two variables (TSS concentration and spectral reflectance of the bands).

#### 2.3.2. Markov Dynamic Evaluation

As an important method to study the state of an event and the law of transition between states, the Markov process can analyze the changing trend of the state at the time of $t_{0}+\Delta t$ by the initial probability of the different states of the event at the time of $t_{0}$ and the probability transfer relationship between states, and can intuitively reflect the dynamic process of an event during this period [45]. It has no after-effects, that is, the state transition probability is only related to the transition start state, the number of transfer steps and the post-transition state. It is independent of the initial moment of the transition [46]. Therefore, the combination of large-scale remote sensing evaluation at a single time and the Markov process method with multi-period stochastic dynamic analysis not only breaks through the limitations of static analysis of traditional remote sensing evaluation but also reflects the trend of dynamic changes in multiple periods. The new research method was provided for the change trend evaluation of TSS concentrations in the Hedi Reservoir area.

To reflect the trend of dynamic changes in the concentration of TSS in the reservoir area in 2014–2018, grids (k) of 30 × 30 m size (equal to the pixel size of Landsat OLI images) are distributed in the reservoir area. Each grid represents a site where every site has a value about the TSS concentration, and each site has a total monitoring of n times in a year. Each site performs multiple monitorings per year based on clear satellite data for the last 5 years. According to Formula (2) in Section 2.2.2, the water quality of the reservoir was classified into 4 levels: levels I, II, III and IV. We set *E* as the above 4 levels, and the corresponding state space can be expressed as E={1,2,3,4} in turn. The Markov process is a time series model, and the construction process is described below [23,35,47]:

Step 1: Establishing the probability transition matrix *P*

The possibility transition in which the water quality changed from *i* level to *j* level can be simulated using an equation as follows:

(5) $$p_{ij}=\frac{n_{ij}}{M_{i}-m_{i}},0\leq p_{i,j}\leq 1;\sum_{i}p_{ij}=1$$

where $p_{ij}$ is the transition probability of level *i* transferred from level *j*, $n_{ij}$ is the number of original data of level *i* transferred from level *j* for n times in the same site of the reservoir, $M_{i}$ is the number of original data sites at level i during the $y_{n}\mathrm{th}$ monitoring and $m_{i}$ is the number of original data sites at level i in the monitoring of *n* times.

Then, the matrix $P={\left(p_{ij}\right)}_{n\times n}$ can be found, and this is the possible transition matrix of the reservoir.

Step 2: Build the progress matrix S

The progress matrix is used to show how to distinguish the water quality deteriorated or improved from *i* level to *j* level in the time of monitoring and it can be calculated using an equation as follows:

(6) $$s_{ij}=(i-j)\left|i-j\right|p_{ij},(i,j\in E)$$

where $s_{ij}$ is the weight which can figure out the extent the water quality deteriorated or improved, and i and j are the levels of water quality that follows as E={1,2,3,4}.

The matrix $S={\left(s_{ij}\right)}_{n\times n}$ is defined as the progress matrix of the probability transition matrix $P={\left(p_{ij}\right)}_{n\times n}$.

Step 3: Simulate the progress degree pd(s)

The progress degree is used to show how much the water quality changed from *i* level to *j* level in the time of monitoring and it can be simulated using an equation as follows:

(7) $$pd(s)=\sum_{i,j}s_{ij}=\sum_{i,j}\left(i-j\right)\left|i-j\right|p_{ij},\left(i,j\in E\right)$$

where pd(s) is the value which shows how much the water quality changed from *i* level to *j* level, $s_{ij}$ is the weight which can figure out the extent the water quality deteriorated or improved and $p_{ij}$ is the transition probability of level *i* transferred from level *j*.

The dynamic evaluation of TSS concentration in the reservoir area is based on pd(s). The pd(s) can enable reflecting on the water quality by ΔL which is the number of levels changed by TSS. At the same time, ΔL is related to TSS, which can intuitively reflect the transfer process of TSS levels. The corresponding relationship among pd(s), ΔL and the transfer process is shown in Table 3. When pd(s) is less than zero, it indicates that the concentration of TSS is increased and water quality is deteriorated; when pd(s) is greater than zero, it indicates that the concentration of TSS in the reservoir area is reduced and the water quality is improved. Besides, different degrees of progress correspond to different concentrations of TSS. If -1<pd(s)<1, the level of TSS has not changed; if 1≤pd(s)<4, the level of TSS is increased by one level; if 4≤pd(s)<9, the level of TSS is increased by two levels; and if 9≤pd(s)<16, the level of TSS is increased by three levels. At the same time, the greater the absolute value of pd(s), the more obvious the difference in TSS concentration, and the greater the degree of improvement or deterioration of the water quality.

Although the progress degree pd(S) depicts the pattern and trend of TSS dynamic changes within one time period, there is no direct relationship to tie the average TSS concentration to the progress degree. For this reason and the convenient comparison of the results, two kinds of TSS concentrations were input to a Markov model to evaluate the dynamic changes in water quality. Among the two kinds of TSS concentrations, one is collected from every pixel in the study area and called pixel-based TSS, and the other is the annual averaged TSS by corresponding to all pixels in this region and called the region-averaged TSS. The evaluation results are composed of the series of TSS grids, meaning that every grid mainly considers the differences in the spatial distribution of the TSS concentration, so that the results of this evaluation are representative throughout the research area.

#### 2.3.3. Accuracy Assessment of TSS Retrieval Model

To further analyze the performances of two kinds of inversion models (band ratio and three-band models) and validate the inversion accuracy, 34 samples were randomly selected from the dataset of 49 samples for the calibration and the remaining 15 samples were used for the validation. Three indicators are used to comprehensively evaluate the pros and cons of the model, including the determination coefficient $\left(R^{2}\right)$, the root mean square error (*RMSE*) and the mean relative error (*MRE*), as in Equations (8) and (9):

(8) $$RMSE=\sqrt{\frac{1}{n}\sum_{i=1}^{n}{\left(y_{i}-y_{i}^{’}\right)}^{2}}$$

(9) $$MRE=\frac{1}{n}\sum_{i=1}^{n}\left|\frac{y_{i}-y_{i}^{’}}{y_{i}}\right|\times 100\%$$

where n represents the number of samples, and $y_{i}$ and $y^{’}{}_{i}$ are the measured values and the model estimates of the TSS concentration, respectively.

## 3. Results

### 3.1. TSS Model

#### 3.1.1. TSS Model Calibration and Validation

As shown in Table 1, the ranges of the TSS data in the Poyang Lake and Hedi Reservoir datasets were similar, whereas TSS and their mean value in Poyang Lake datasets displayed a higher mean value. Therefore, considering the statistical characteristics of the TSS datasets and the wide applicability of the TSS model, this study used all the datasets from different regions to calibrate and validate the TSS remote sensing estimation model. The aggregated data (a total of 49 pairs of valid data) were divided into two datasets, among which 34 pairs (approximately 70% of the total number of samples) were used to calibrate the TSS retrieval model and the rest of the dataset (15 samples, approximately 30% of the total number of samples) contained the validation data.

According to the method in Section 2.3.1, the empirical band ratio and three-band retrieval models with the highest coefficient of determination were obtained through regression analysis among different band combination forms that estimate the TSS concentration. The band ratio and three-band retrieval models are as follows:

(10) $$\mathrm{TSS}=172.191\times \ln^{2}[R_{rs}(b3)/R_{rs}(b4)]-190.809\ln[R_{rs}(b3)/R_{rs}(b4)]+61.6$$

(11) $$\mathrm{TSS}=-16.517\times \ln[[R_{rs}{}^{-1}(b_{4})-R_{rs}{}^{-1}(b_{3})]R_{rs}(b_{2})]-8.363$$

where $R_{rs}(b_{2})$, $R_{rs}(b_{3})$ and $R_{rs}(b_{4})$ represent the reflectance of OLI data in the blue band, green band and red band, respectively. The unit of TSS concentration is in mg/L.

**Figure 2:**
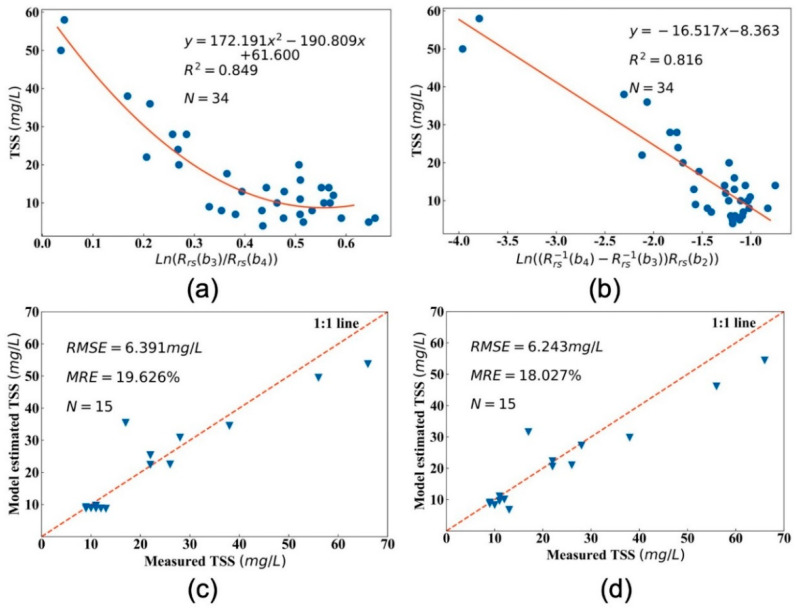
The calibration and validation of the TSS remote sensing estimation model between simulated OLI-based $R_{rs}$ and in situ measurement data are shown in the figure, where (**a**) shows the model calibrated by the band ratio algorithm, and (**b**) shows the model of the three-band algorithm. Plots of measured vs. model-estimated TSS in Hedi Reservoir with a 1:1 fit line (red dotted line), where (**c**) is the result of validating the band ratio model, and (**d**) is the validation result of the three-band model.

Figure 2 shows the different TSS retrieval models based on the best combination of band reflectance, where (a) is the band ratio model and (b) is the three-band model. At the same time, this study also compares the measured TSS values with the predicted values by different models and draws a scatter plot (as shown in Figure 2c,d).

For the term of the two retrieval models, we find the best regression relationship between simulated OLI-derived $Ln(R_{rs}(b_{3})/R_{rs}(b_{4}))$ or $Ln((R_{rs}{}^{-1}(b_{4})-R_{rs}{}^{-1}(b_{3}))R_{rs}(b_{2}))$ and measured TSS data with a higher coefficient of determination (0.849 in the band ratio model and 0.816 in the three-band model). The results show that the two models have a better performance, where *R^2^* is 0.849 and 0.816, respectively, indicating that both models can be used to estimate the TSS concentration in this study area. The *RMSE* of the three-band model is 6.243 mg/L, which is 2.31% lower than the 6.391 mg/L of the band ratio model. The *MRE* of the three-band model (19.626%) is smaller than the band ratio model (18.027%), and both are less than 20%. Although the determination coefficient of the band ratio model is 4% higher than that of the three-band model, the validation of the band ratio model is higher than that of the three-band model. The *RMSE* of the band ratio model is 0.148 mg/L higher than that of the three-band model, and the *MRE* has increased by 8.87%. At the same time, the scatter plots of the TSS estimated and the actual values of TSS are well distributed around the 1:1 line. Generally speaking, using the first-order three-band model (as in Equation (11)) for Hedi Reservoir simplified the simulation of the model calibration and validation ($R^{2}=0.816,RMSE=6.243\mathrm{mg}/L,MRE=18.027\%$) compared to the second-order band ratio model (as in Equation (10), $R^{2}=0.849,RMSE=6.291\mathrm{mg}/L,MRE=19.626\%$) with similar performance.

#### 3.1.2. Comparison and Verification of TSS Models

Several empirical and semi-analytical algorithms have been proposed to estimate TSS concentration, from clear open sea waters and slightly turbid coastal waters to highly turbid inland waters. However, the performance of different models cannot be directly compared because of the differences in band positions and bandwidth designs between Landsat and other sensors, such as MODIS and MERIS. Taking these factors into account, Table 4 lists representative TSS retrieval algorithms of the same type of sensor to facilitate the comparison and verification of the performance and accuracy of the model. Here, we were able to use the simulated $R_{rs}$ and field measurement data from the aggregated data in Table 1 to validate these models, in which the calibration dataset and the verification dataset were used to recalibrate the model parameters and validate the accuracy of all chosen models, respectively. The model algorithm and verification results are shown in Table 4.

The validation results shown in Table 4 indicate that the average relative errors between the estimated and measured values of these models were 47.28%, 31.28%, 27.36%, 63.54%, and 34.16%. The accuracies of those models were low and could not meet the requirement of accuracy for TSS estimation. Contrarily, the proposed model calibrated in this study (whether it is a band ratio model or a three-band model) has the highest accuracy. The *RMSEs* and *MREs* of all validation data for the band ratio model are 6.39 mg/L and 19.62%, and 6.24 mg/L and 18.02% for the three-band model. The two models calibrated in this paper have the best validation accuracy among all models. Besides, the determination coefficients in the band ratio model and three-band model are 0.84 and 0.81, respectively, thus these models can still explain the TSS concentration variation more than 80%. In summary, compared to the second-order band ratio model, the first-order three-band model may better suit estimating the TSS concentration in Hedi Reservoir. 

#### 3.1.3. Accuracy Assessment Based on Synchronous Remote Sensing Images

**Figure 3:**
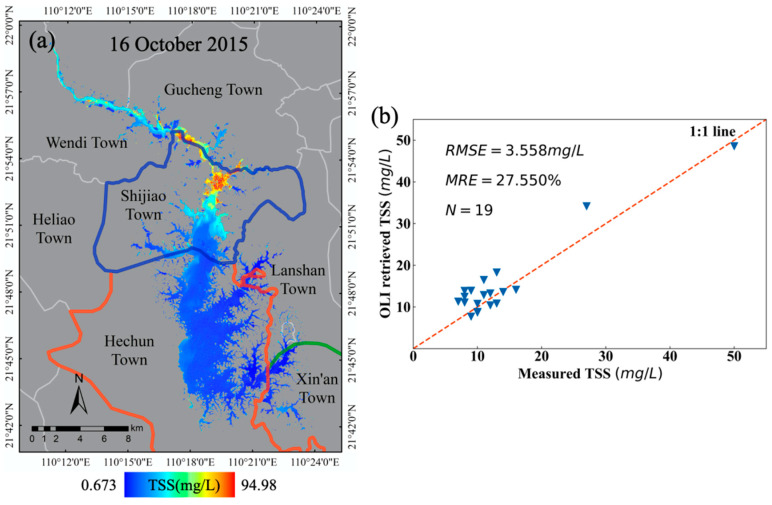
Estimated TSS concentrations based on the three-band model from Landsat OLI imagery in Hedi Reservoir on 16 October 2015, where the different color lines stand for towns over the reservoir basin (**a**), and comparison between the in situ measured and OLI imagery-retrieved TSS concentrations (**b**). The color scale is the legend of the TSS concentrations, in mg/L.

Besides, we also used 19 in situ data to validate the three-band model on the synchronized Landsat OLI imagery. The validation result shows that the *RMSE* and *MRE* between 19 field TSS concentrations are 3.55 mg/L and 27.55%, respectively. Besides, all the sample points can be evenly distributed around the 1:1 line and the error is within 30%. Figure 3 shows the retrieval and validation results of the TSS concentration in Hedi Reservoir on 16 October 2015, where (a) depicts the spatiotemporal distribution pattern of the TSS concentration in Hedi Reservoir with a large variation ranging from 0.67 to 94.98 mg/L and (b) shows the validation results between in situ measured TSS and OLI-retrieved TSS. 

### 3.2. Spatiotemporal Characteristics of TSS Concentration

#### 3.2.1. Analysis of Optical Characteristics of the Water Body in Hedi Reservoir

**Figure 4:**
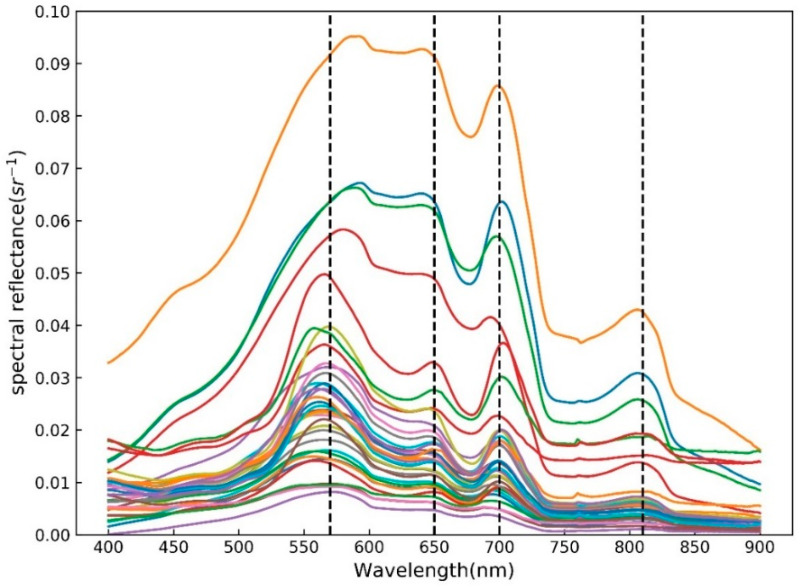
A number of 35 measured spectra of typical water collected by ASD in the 2015 cruise over Hedi Reservoir, where the different color lines represent the different spectra of 35 water samples.

The maximum spectral reflectances measured in the field are all less than 0.1 (Figure 4). The spectral curve of the water body of Hedi Reservoir shows a significant four-peak trend different from coastal and estuarine waters [18]. The spectral curve corresponding to the change in the concentration of TSS and other watercolor components also shows different changes at each peak. In the range of 560–590 nm, the first strong reflection peak appeared due to the weak light absorption and cell scattering of chlorophyll in phytoplankton [48]. The reflectance corresponding to the peak area gradually increases as the concentration of TSS increases. At the same time, since cyanobacteria are the absolute dominant species in the water body of Hedi Reservoir [30,49,50], the cyanobacteria have an absorption peak near 620 nm, therefore reflectance appears as an absorption valley or shoulder-shaped near 630 nm [48,51,52]. Similarly, a reflection valley appears near 675 nm due to the strong absorption of algae chlorophyll in the red light band [51]. At the same time, a clear reflection peak was generated between the two reflection valleys (near 620 and 675 nm), and the peak was located near 650 nm, which is one of the important optical signals to characterize whether the water body contains phycocyanin [26,53]. Besides, the reflectance of the water body corresponding to this peak will increase with the increase in the TSS concentration, and its position will also move along the direction of the longwave, which will appear as a “red shift” phenomenon [48,54], which will result in a wide range of the peak wavelength coverage. The third reflection peak caused by the Raman effect appears around 700 nm [55], which is the most prominent spectral feature of algae-containing water and is considered to be an important basis for determining whether the water contains algae chlorophyll. Finally, the reflection peak around 810 nm is an important spectral feature of the presence of suspended inorganic matter in water, and it can be used to distinguish lower and higher TSS concentrations. The water body of Hedi Reservoir has mainly suspended particles, showing strong backscattering. With the increase in the concentration of TSS, the characteristic of the above four bands are more prominent, resulting in higher reflectance of reservoir water. However, in the near-infrared region, the backscattering coefficient of pure water gradually decreases with the increasing wavelength [56,57]. On the other hand, the strong absorption of water in the near-infrared region [56] leads to a generally low reflectance of water.

#### 3.2.2. The Temporal and Spatial Patterns of TSS Distribution in Hedi Reservoir

**Figure 5:**
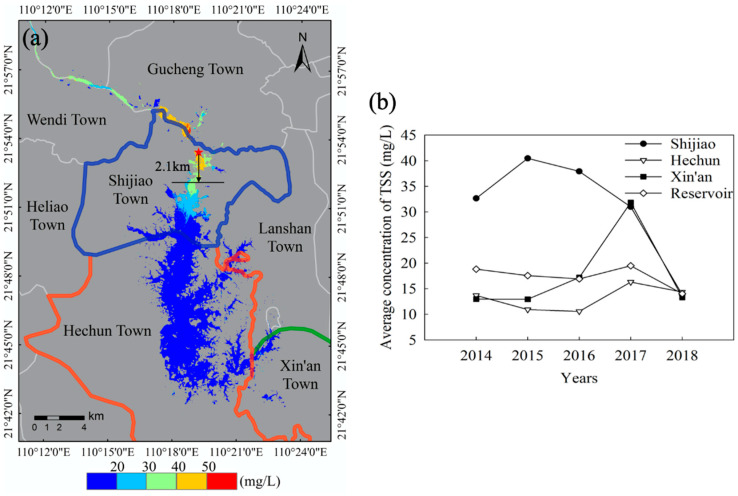
The spatial distribution of TSS concentration based on OLI imagery in Hedi Reservoir, where different colored lines represent different boundaries of towns: (**a**) mapping of the averaged TSS concentration in Hedi Reservoir from 2014 to 2018, and (**b**) the comparison of the annual average TSS concentration in different areas within the reservoir. The color scale is the legend of the average TSS concentrations, in mg/L. The location of the red star in the picture is the government of Shijiao Town.

The spatial and temporal distribution of the concentration of TSS in the reservoir area was studied by applying the three-band model to satellite imagery and plotting the temporal and spatial distribution of TSS in Hedi Reservoir. Based on the results of TSS inversion in the long-term sequence of the reservoir area (2014–2018), the average concentration of TSS and spatial pattern distribution in the reservoir area were analyzed and compared (Figure 5).

Figure 5a reflects the distribution of the average concentration of TSS in the reservoir area during the five years, which is “High in the upper reaches of the Hedi Reservoir and low in the downstream”, and the upstream concentration of TSS reaches 2.5 times in the downstream. From 2014 to 2018, the concentration of TSS in the reservoir area was between 7.92 and 60.67 mg/L, with an average of 16.06 mg/L. The average concentration of TSS in the reservoir area of Shijiao Town was 31.54 mg/L, and the concentration above 40 mg/L was farthest away at 2.1 km toward the south of Shijiao Town. As shown in Figure 5b, the quality of water in the reservoir area was stable in 2018, and its average concentration of TSS has remained at 14–20 mg/L. The concentration of TSS in the Hechun Town area is consistent with the changing trend of the whole reservoir area. On the contrary, the concentration of TSS in Shijiao Town changed significantly from 2014 to 2018 and reached a maximum of 40.46 mg/L in 2015. Until 2018, the concentrations returned to the normal level of the reservoir area (13.84 mg/L). The water quality in the study area was mainly influenced by the water in the Shijiao area where the TSS were the highest.

**Figure 6:**
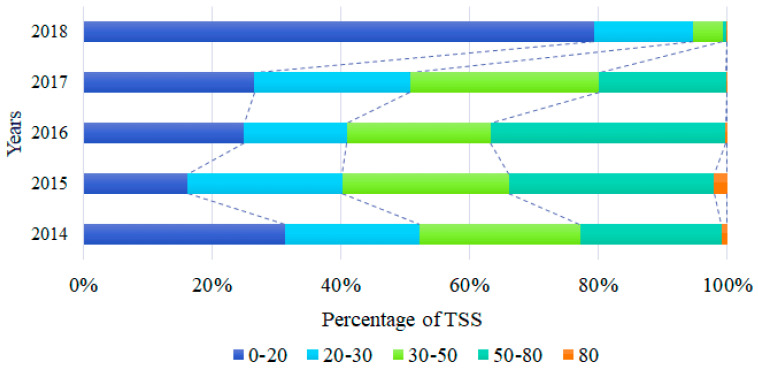
Percent distribution of annual average concentrations of TSS in Shijiao Town from 2014 to 2018, including levels with thresholds of 20, 30, 50 and 80 mg/L.

To further reveal the temporal and spatial variation in the water quality in Shijiao Town, the average TSS gradient map in the region was analyzed at intervals of 20, 30, 50 and 80 mg/L (Figure 6). Overall, the average concentration of TSS in Shijiao Town was 31.54 mg/L between 2014 and 2018. In the first two years, the concentration of TSS increased and gradually spread downstream. The area of concentrations ranging from 50 to 80 mg/L was increased by 66.42%, accounting for 31.84% of the reservoir area of the region. On the contrary, in the last two years, the average concentration of TSS in Shijiao Town decreased to 22.41 mg/L, and the areas of 30–50 and 50–80 mg/L decreased by 84.48% and 96.88%, respectively. Besides, the area of the 0–20 mg/L range increased from 26.56% to 79.32%, and each gradient concentration distribution changed significantly. In the five years, the annual average concentration of TSS in the water body of Shijiao Town reached a maximum of 40.46 mg/L in 2015, and it reached the lowest value in 2018 and fell to 13.84 mg/L, which decreased by 65.79% and reached the first-level water quality standard. In fact, since September 2017, the local government has fully implemented a strong work plan for the river chief system, reflecting the good effectiveness of national policy implementation.

### 3.3. Analysis on Driving Factors of TSS Change

#### 3.3.1. Changes Characteristic of the Concentration of TSS in Flood Season and Dry Season

**Figure 7:**
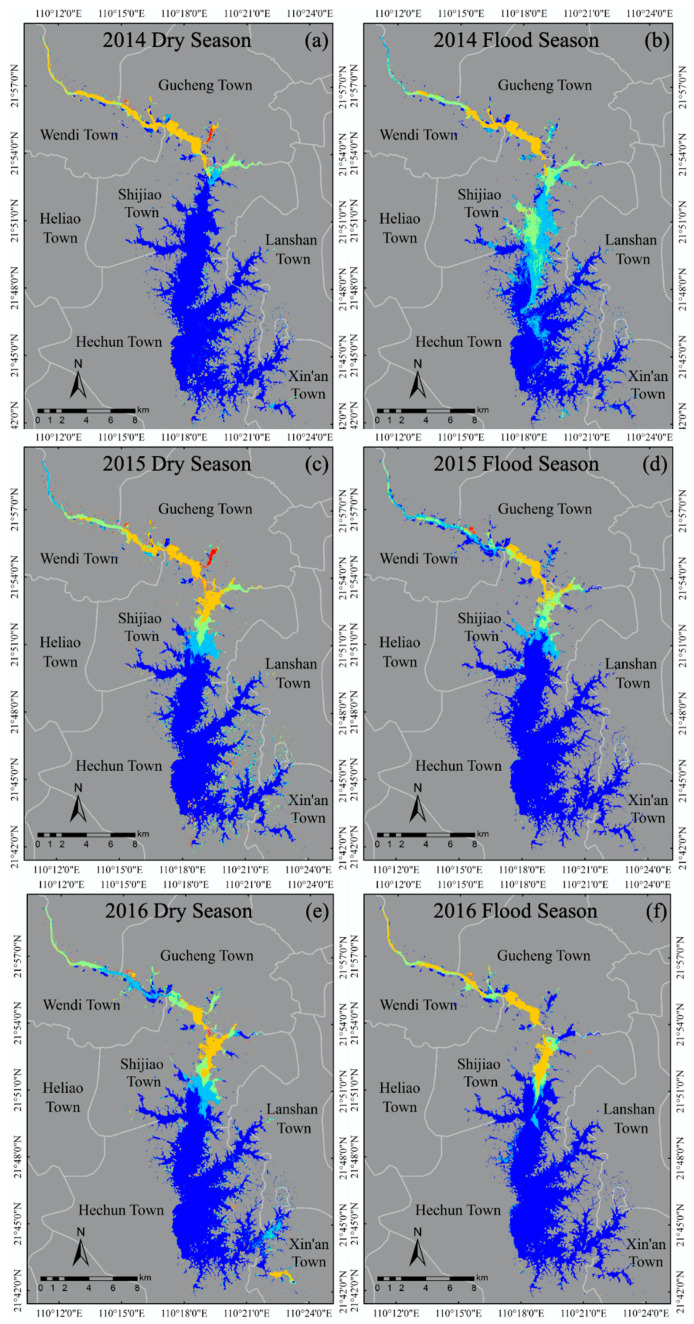

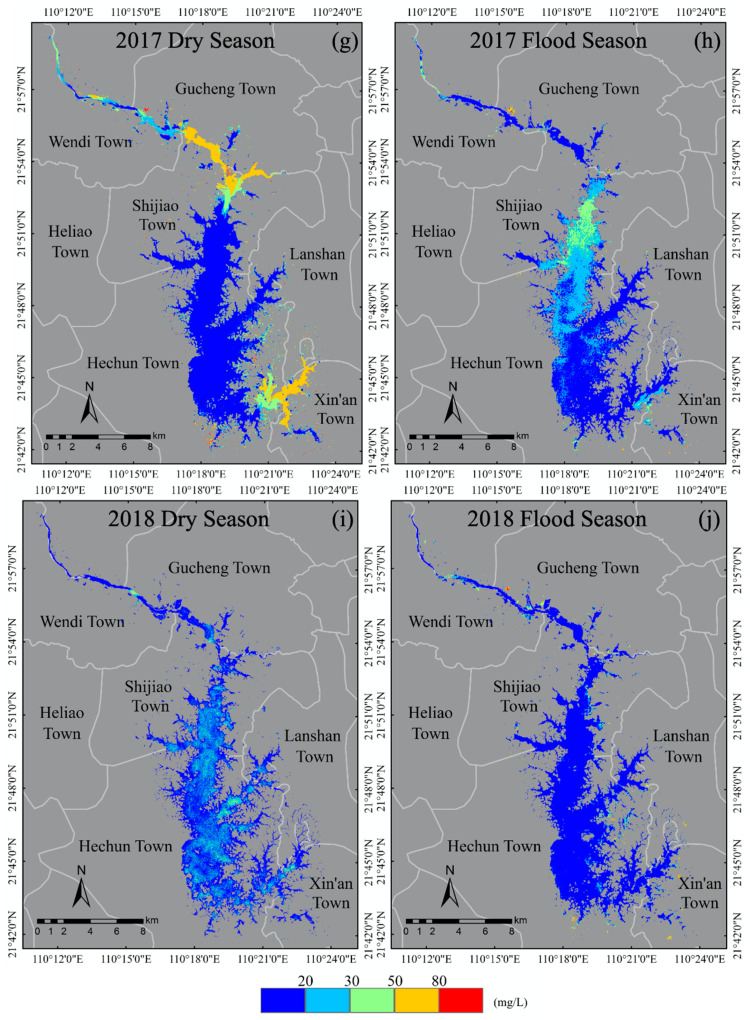
The spatial distribution map for the estimated concentration of TSS on an annual average in dry and flood seasons of Hedi Reservoir from 2014 to 2018 is mapped as shown in the figure, among which (**a**,**c**,**e**,**g**,**i**) are in the dry season within 5 years, and the rest (**b**,**d**,**f**,**h**,**j**) fall into the flood season. The color scale is the legend of the average TSS concentrations, in mg/L.

**Figure 8:**
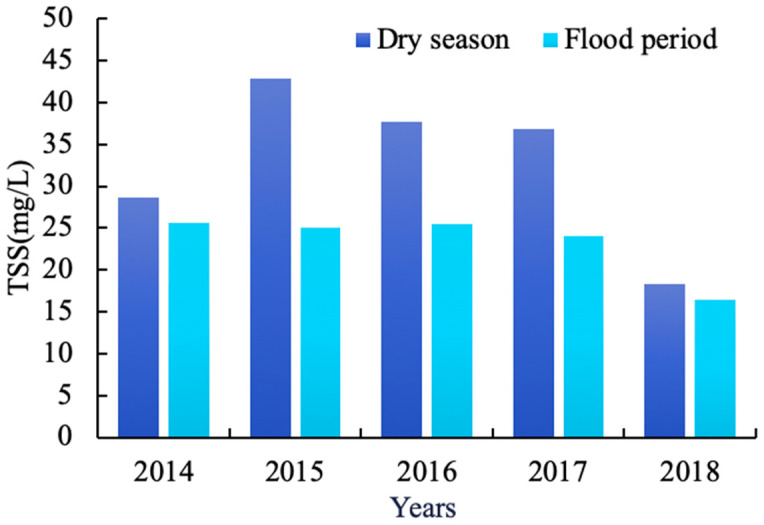
Comparison of annual average TSS concentrations in the reservoir area of Shijiao Township from 2014 to 2018 in different seasons.

In addition to the overall analysis of the concentration of TSS in the reservoir area for the five years, the temporal and spatial variation characteristics of TSS in the reservoir area were analyzed by the dry season and flood period, and we produced spatial distribution maps in different seasons (Figure 7). It can also be observed from Figure 5b that water quality in the Hechun Town area is good during the five years, and the averaged concentration of TSS is stable at about 13.16 mg/L. Therefore, the Shijiao Town area was taken as the key research object and analyzed in depth. Comparing the changes in the concentration of TSS in Shijiao Town in different seasons (Figure 8), in general, the concentration of TSS in the dry season with an average of 32.86 mg/L was higher than that in the flood period (23.45 mg/L). Especially in 2015, there was the largest difference of 17.8 mg/L in the average TSS concentration for dry and flood seasons. Within the five years, the differences in averaged TSS between dry and flood seasons were 9.52 mg/L. Despite some increase in the dry season before 2015, the TSS concentration decreased by 24.58 m/L, from 42.86 in 2015 to 18.28 mg/L in 2018. On the contrary, during the flood period, the TSS concentration maintains about 25 mg/L in the three former years and then decreases to 16.45 mg/L in 2018.

#### 3.3.2. Effect of Precipitation on the Concentration of TSS

Normally, precipitation may generate some effects on water quality, where especially heavy rain and the surface runoff can flow into the lake or reservoir and produce a turbid phenomenon in the water body, even posing a threat to the local water environment. 

**Figure 9:**
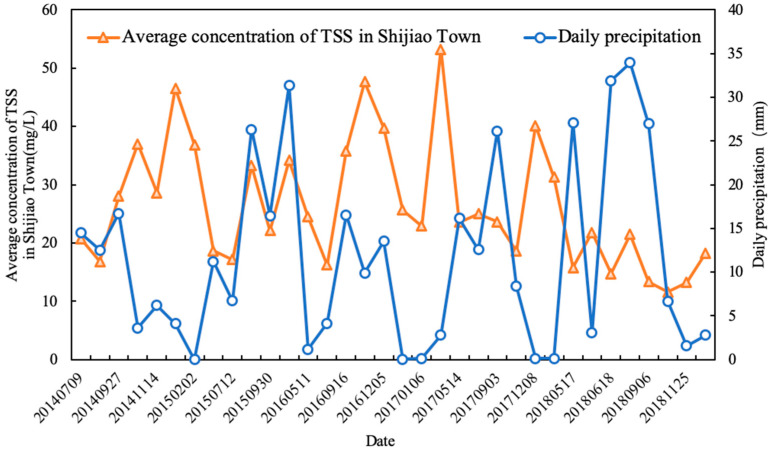
Comparison of time series variation of TSS concentration and daily precipitation in Shijiao Town, where the average TSS concentration is signed by orange triangles and the daily precipitation is signed by blue dots.

In the five years, the TSS concentration changes are smaller in the whole reservoir area. As we can see from the results shown in Figure 5b, the concentrations of TSS in Hechun Town and Xin’an Town near the dam were below 20 mg/L (13.16 and 17.64 mg/L, respectively). However, the temporal and spatial distribution of upstream Shijiao Town changed significantly and the average concentration of TSS was as high as 47.67 mg/L. Besides, because Shijiao Town is located at the junction of Guangdong and Guangxi Provinces, it has accepted the pollution from upstream mining and frequent human economic activities, which seriously impacts the water quality of the area. Therefore, taking this typical area as an example, combined with precipitation and referring to the level of precipitation (GB/T 28592-2012, published by Standardization Administration of China), we analyzed the feature of TSS change. In practice, we obtained the accumulated rainfall data of two weeks before the date of satellite imaging and used the average value as the daily precipitation. Taking the acquired multi-source satellite imagery of the five years as a time series, the daily precipitation and the trend of the corresponding concentration of averaged TSS in the region are plotted (Figure 9). Obviously, during the dry season, the daily precipitation is negatively correlated with the change in the TSS concentration within the reservoir area of Shijiao Town. The concentration of TSS in this area decreases with the increase in daily precipitation. It is not difficult to find that, in Figure 9, the rainfall decreased from 17.90 on 27 September 2014 to 3.87 mm 13 October 2014, but the TSS concentration increased from 27.98 to 36.97 mg/L from strong mankind activity for same period. However, in the flood period, the daily precipitation change is consistent with the trend of the TSS concentration, which is positively correlated. From Figure 9, the rainfall increased from 7.17 on 12 July 2015 to 28.31 mm on 14 September 2015, while the TSS concentration increased from 17.17 to 33.25 mg/L. In fact, suspended matter in water shows a slow settling process [58]. Due to the influence of different rainfall intensity, the settling process of TSS will change, and the TSS concentration will produce different variations. When the rainfall intensity is small, especially in the dry season, the concentration of TSS in water from mankind activity of upstream is greater than the resuspension rate caused by rainfall, so the TSS concentration is negatively correlated with rainfall during the dry season. On the contrary, the rainfall intensity in the flood season is greater, and the settling rate of TSS in water is less than the resuspension rate caused by rainfall, so the TSS concentration increases with the rainfall intensity.

#### 3.3.3. The Influence of Human Activities on TSS Concentration

**Figure 10:**
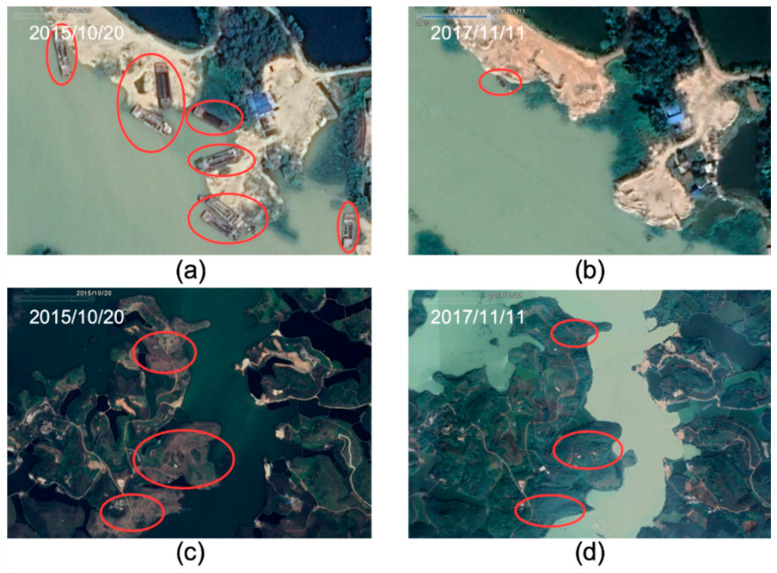
Historical view of the Google Earth image showing the polluted water environment in Hedi Reservior caused by different anthropogenic activities: (**a**) shows the status of large-scale illegal sand mining activities before the river chief system was implemented, (**b**) indicates that sand mining activities are effectively restricted after the execution of the river chief system policy and (**c**,**d**) depict the extensive area of eucalyptus planting with frequent cutting. All the red circled areas in Figures (**a**,**b**) indicate changes in sand mining vessels in the same area of the reservoir before and after implementation of the river chief system policy; (**c**,**d**) reflect the changed progress in eucalyptus planting and cutting.

On the whole, due to the Shijiao Town being located in the middle reaches of the Jiuzhoujiang River and upstream of Hedi Reservoir, it is an important import source of water pollution in Hedi Reservoir from the upstream of the watershed. The water the Shijiao area generates has severe impacts on the water quality of the whole reservoir and the concentration of TSS there is the highest compared with other areas in Hedi Reservoir. According to the consequences of this study, there are two reasons to exemplify this phenomenon. On the one hand, Shijiao Town is located across the boundary of Guangdong and Guangxi Provinces and is affected by the water quality of the upstream Jiuzhoujiang River. On the other hand, field investigations and related research have shown that illegal sand mining activities in the region are frequent and the concentration of TSS is subject to the effect of large suspension. In 2016, the concentration of TSS in Xin’an Town began to increase, and the concentration of TSS reached 31.84 mg/L and then decreased to a normal level in 2018. After a historical view of the Google Earth image and field investigation (shown in Figure 10), it was found that there were large areas of eucalyptus planting and large-scale illegal sand mining activities in the area. Besides, eucalyptus has great economic benefits, and people are also exerting a subtle influence on the surrounding water environment while pursuing the economic benefits of eucalyptus. Particularly, eucalyptus deforestation and fertilization periodically have a serious impact on water quality [59], which is also an important reason for the sudden increase in the TSS concentration in Xin’an Town in 2016.

Consequently, to further reveal how anthropogenic activities, especially the illegal sand mining activities, affect the concentration of TSS, this research also compared the differences in TSS in different seasons under such human interruption. The frequent sand mining activities in Shijiao Town make the concentration of TSS distinctly higher than that during the flood season. The reason is that the water level in the dry season is lower and affected by the large need for sand in the market, which is more conducive to the collection of sand for the period. The sand mining activity causes the TSS of the water in the reservoir area to resuspend, which promotes the increase in the concentration of TSS, accompanied by the decrease in water transparency. Just as the research in China’s four largest freshwater lakes also draws similar conclusions, the concentration of TSS becomes high in the dry season and low in the flood period after sand dredging activities begin [7,37].

Despite the fact that human-induced changes severely threaten the local water environment to bring about the problem of soaring TSS, the natural conditions, especially precipitation and seasons, also cause identical effects on TSS and they become the most significant factor to reveal the dynamic changes in TSS. Regularly, there is a significant seasonal variation in the effect of precipitation on the concentration of TSS. The TSS concentration in the upstream (Shijiao Town) is more affected by rainfall in the dry season with an average TSS of 33.66 mg/L than in the flood period with an average of 22.45 mg/L. The reason for this phenomenon can be attributed to the fact that the catchment in Shijiao is the TSS deposition area produced by the upper river discharge and the surrounding load runoff. This unavoidable phenomenon, where the TSS concentration before a strong rainstorm in the dry season is lower than the one in the rainy season, was also found in the study by Chen et al [31]. 

The changing law of TSS depends on the two aspects of natural conditions and human activities. There is no doubt that the great changes in water quality in the study are inseparable from the extensive planting and frequent felling of eucalyptus trees, in addition to the cultivation of fruit trees, which are very common in western Guangdong and Guangxi. Considering the specific influence factors on water quality, the TSS in Hedi Reservoir shows obvious differences under different seasons and precipitations. At the upstream reservoir area in Shijiao Town, the TSS is higher in the dry season from upstream mankind activity, especially in 2015 with an average TSS of 42.85 mg/L, and presents a negative correlation with rainfall intensity; on the contrary, the TSS is lower during the flood period, especially in 2015 with an average of 25.06 mg/L, and the TSS trend is positively correlated with rainfall intensity. As a typical trans-provincial area of Hedi Reservoir, there is the largest difference in the average TSS concentration of 17.794 mg/L between the flood and dry seasons in 2015 in Shijiao Town, while the smallest difference of TSS in 2018 was 1.831 mg/L (Figure 8). The concentration above 40 mg/L was farthest away at 2.1 km toward the south of Shijiao Town.

At the same time, we also found that, in the dry seasons of 2016 and 2017, the downstream reservoir area (southeast) near Xin’an Town also experienced deterioration in water quality with an average TSS of 24.50 mg/L due to the cultivation of orchards. Besides, since the implementation of the river chief system from September 2017, the water quality has been significantly improved in the upstream reservoir area (north) of Shijiao Town, where the average TSS concentration in Shijiao has been reduced from 30.98 in 2017 to 13.84 mg/L in 2018 (Figure 5b). At the same time, the average TSS concentrations in Hechun Town, Xin’an Town and the entire reservoir all decreased from 16.28, 31.84 and 19.47 in 2017 to 14.35, 13.29 and 14.01 mg/L in 2018, respectively, which indicates the regional effectiveness of implementing the river chief system of China.

### 3.4. Markov Evaluation of TSS Dynamic

**Figure 11:**
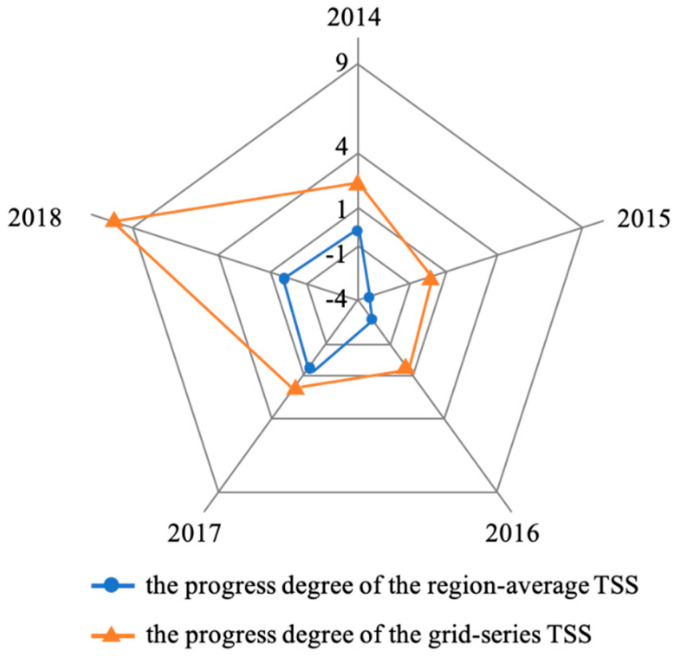
The evaluation results of the progress degree of TSS in the Shijiao Town area of Hedi Reservoir from 2014 to 2018. The blue part is based on the region-averaged TSS concentration in the Shijiao Town area, and the orange part is based on the pixel-based TSS concentration in the Shijiao Town area.

According to the results of the TSS concentration changes in Hedi Reservoir from 2014 to 2018, except for the water body in Shijiao Town, the concentration of the TSS in other areas has been relatively stable at the level of I. Therefore, this study focuses on the Shijiao Town waters in the high-risk area of TSS as the key monitoring object and quantitatively evaluates and analyzes the dynamic change process of the TSS in this area according to the method in Section 2.3.3. The results of the progress degree pd(S) for two kinds of TSS in the reservoir area from 2014 to 2018 are as follows in Table 5. The whole results that reflect the progress degree of TSS in Shijiao from 2014 to 2018 can be seen in Figure 11.

Figure 11 illustrates changes in the TSS progress degree by using the Markov evaluation model and applying two kinds of TSS for that period (from 2014 to 2018). Obviously, in 2018, the progress degree of the pixel-based TSS recorded the highest value, at 10.232, followed by 2014, 2017, 2016 and 2015, with 2.493, 2.042, 0.819 and 0.194, respectively. On the whole, from 2014 to 2018, the progress degree produced by the pixel-based TSS is greater than 0, indicating that the water quality has been improved, and it includes all possible states in the process of water quality improvement. This maximum progress value is 10.23 calculated from the pixel-based TSS of each grid in Shijiao Town, which indicates that the concentration level of TSS has spanned three levels. Similarly, the progress degree produced from the region-averaged TSS in Shijiao Town showed a minimum value (−3.333) in 2015 and a maximum value (0.899) in 2017, followed by 2016 (−2.667), 2014 (0) and 2018 (0.667). However, except for 2014 and 2018, most results of the average TSS are below 0, which represents the deterioration of the water quality. The result of the regional average TSS progress degree recorded at −3.333 in 2015 stated that the water quality had deteriorated and the TSS concentration level had dropped by one level.

According to the evaluation results shown in Figure 11, it can be seen that whether or not the progress degree from the pixel-based TSS or the region-averaged TSS in Shijiao Town was simulated, the above two types of data reach the lowest in 2015. At the same time, the results obtained from the two different types of TSS data have roughly the same trend shown by the red and blue lines in Figure 11, which confirms the validity of the Markov assessment method proposed in this study. The progress degree of the pixel-based TSS in 2014 was higher than in 2017 (2.493 vs. 2.042). On the contrary, different from the pixel-based TSS, the progress degree of region-averaged TSS in 2014 was less than in 2017 (0 vs. 0.899). The difference in the progress degree of the pixel-based TSS (2.493 in 2014, 2.042 in 2017) and the regional average TSS (0 in 2014, 0.899 in 2017) is mainly due to the spatial distribution variance. Whether the progress of the pixel-based TSS or the regional average TSS between 2014 and 2017, the number of levels changed by TSS still stays the same which is 1 and 0, respectively, according to Table 3.

**Figure 12:**
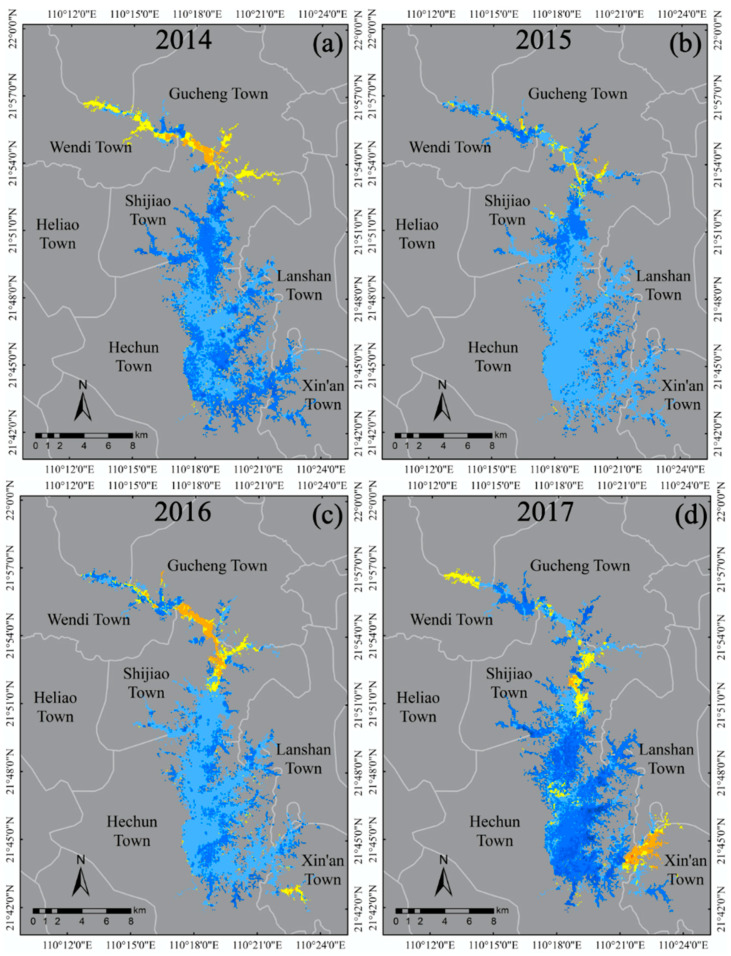

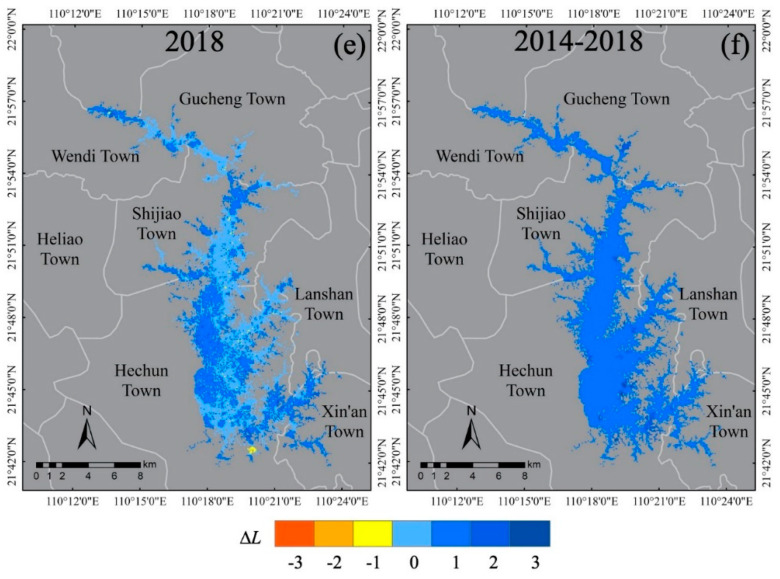
The spatial distribution map of water quality variation calculated by the progress degree combining the Markov model and the remote sensing method from 2014 to 2018 in Hedi Reservoir, where (**a**–**e**) show the annual spatial variation distribution based on the progress degree and (**f**) presents the spatial variation within the whole five years. The color scale marked as ΔL indicates the difference in the number of changes in the two TSS levels during the change in the TSS level status. It means that the TSS level has increased and the water quality has improved when ΔL is greater than 0, otherwise the TSS level has decreased and the water quality has deteriorated. Especially, ΔL is 0, which means that the TSS level has not changed and the water quality remains stable.

To intuitively reflect the detailed change process of the large-scale dynamic in water quality, this study combines the remote sensing method and Markov model into mapping the spatial-temporal distribution of water quality changes in Hedi Reservoir from 2014 to 2018 (in Figure 11). Specifically, the progress degree calculated by the method in Section 2.3.3 is used to represent the change in water quality. It can be seen that the progress degree of water quality in the upstream reservoir is relatively low, which was −8.236 in 2014, −7.851 in 2015 and −8.632 in 2016. However, since 2017, lower progress degree levels of the downstream reservoir near the shore and in some bays have been found, which were −10.346 in 2017 and −3.448 in 2018. According to the statistics of the progress of the reservoir area from 2014 to 2018, it was found that the parts with the progress degree at more than 1 accounted for 46.2%, 22.17%, 22.33%, 59.28% and 49.45% and show an increasing trend. The part where the progress degree is at more than 1 indicates that the TSS concentration has decreased and the corresponding ΔL can be ensured according to the color scales in Figure 12. In contrast, the progress degree which is less than −1 means that the TSS concentration has increased, and the corresponding proportions account for 10.08%, 3.04%, 9.28%, 11.43% and 0.63%. In particular, the proportion of water quality deterioration decreased by 94%, from 11.43% in 2017 to 0.63% in 2018. It can be seen from Figure 12f that the progress within these five years (from 2014 to 2018) is between 1.219 and 10.771, which means that the TSS concentration has not increased and the water quality has improved during this period.

## 4. Discussion

Traditional water quality cross-section monitoring is limited to detecting in small-scale with high time consumption and high cost. Considering the advantage of Markov and remote sensing, a novel water quality evaluation method is proposed, which breaks through the traditional water quality cross-section point sampling and detecting. This Markov-based water quality remote sensing evaluation method can directly associate the TSS concentration level with the degree of progress to quantify the dynamic change process of the TSS in multiple time series, which has great advantages especially in evaluating the implementation of the river chief system compared with the traditional water quality evaluation method. It can potentially be implemented in other developing countries in the future. The study reveals the dynamic continuous trend of TSS in the five years. Especially in 2018, the degree of progress is 10.232 with the relatively largest improvement. Additionally, the progress degree produced from the Markov model can be related directly to the change in TSS levels, which further indicates the meanings or effects of water quality status or change for corresponding management measures taken. At the same time, this method constructs a continuous, integrated and dynamic spatial-temporal pattern in the analysis of TSS, and driving factors were studied to effectively find the problems of point and non-point source pollution in the reservoir area. This is of great significance for the real-time monitoring, future prediction and management of the aquatic environment in the reservoir or other areas. The method presented will have great potential for dynamic analysis of TSS in big data scenarios of remote sensing images.

The spectral curve of the water body of Hedi Reservoir shows a typical four-peak pattern that probably results in the unsuitability of the previous band ratio model in coastal and inland waters [10,18,24,29,43]. Part of the reason can be attributed to the abundant phytoplankton and the majority of cyanobacteria in the water body of Hedi Reservoir [49,50]. A semi-empirical three-band TSS retrieval model (Equation (11)) was developed under the consideration of the special four-peak optical characteristics of water bodies ($R^{2}=0.816,N=34$). The three-band model has better validation accuracies (RMSE=6.24mg/L,MRE=18.02%,N=15), which can be compared to the accuracies of previous Landsat-based models in Table 4 (RMSE:8.78–20.57mg/L,MRE:27.36%–63.54%, [18,22]). It has also obtained reasonable validation accuracy (RMSE=3.55mg/L,MRE=27.55%,N=19) through 19 in situ data and synchronized Landsat OLI images. Simultaneously, the TSS retrieval models in nearby coastal waters in South China commonly studied by the previous researchers do not apply to inland water bodies, as the phytoplankton types in estuaries and coasts are different from reservoirs such as diatoms or cyanobacteria. Additionally, the TSS three-band model proposed in this study also includes the 20 pairs of field data in Poyang Lake, which increases the applicability of the model. To a large extent, the good performance of the three-band model developed here can provide a scientific reference for water quality remote sensing in South China.

There are some uncertainties for this study in the inversion of water quality parameters, which are mainly reflected in the following aspects. On the one hand, the accuracy of atmospheric correction for remote sensing imageries plays a decisive role in the reliable retrieval of optical parameters of inland waters. There are differences in the spectral reflectance resulting from the different parameter settings in the same atmospheric correction model or from using different atmospheric correction models, which may affect the accuracy of the retrieval optical parameters of inland water bodies. On the other hand, the time difference in the in situ measurement data as another important factor might introduce some uncertainty, which might interfere with the application and performance of the retrieval model. Most importantly, there might be differences over the component of the water body (including bio-properties) influenced by natural factors in different seasons. In summary, the above uncertainties are worthy of continued attention in future research.

Since the implementation of the river and lake chief systems and other ecological civilization policies by the local government from September 2017, the sand mining activities distributed in Shijiao Town (bordering Guangxi) have been restricted and it is not difficult to find that the number of sand mining vessels has been significantly reduced from Google images (Figure 10a,b). Most importantly, it revealed that the water quality of Hedi Reservoir has been significantly improved. The average concentration of TSS was reduced from 32.65 in 2014 to 14.02 mg/L in 2018. The decrease in TSS reflects the implementation effect of the river and lake chief system by the Chinese government. However, the water environment of Hedi Reservoir is still subject to potentially serious threats from upstream turbid runoff from Guangxi Province. In addition to TSS, we should continue to pay attention to the high-frequency remote sensing analysis of more water environment parameters in the future (such as total nitrogen, total phosphorus and ammonia nitrogen) in drinking water sources such as Hedi Reservoir.

## 5. Conclusions

The water quality assessment method combining Markov and remote sensing realizes the innovation of large-scale spatial monitoring across administrative boundaries instead of traditional cross-sectional monitoring. The degree of water quality improvement simulated by the Markov model can intuitively reflect how much water quality has improved or deteriorated, and can understand how the TSS level has shifted spatially. It also reflects the changes in water quality by the trend analysis of the Markov model since the local implementation of the river chief system of China in 2016.

Taking Hedi Reservoir in the trans-provincial basin of South China as an example, a specific three-band TSS model for the inland reservoir in Guangdong Province of South China was developed ($R^{2}=0.81,\;\;N=34,p\;\mathrm{value}<0.01$) based on in situ spectra and Landsat OLI imagery, which is the first such study of an inland reservoir available in Guangdong or South China, where there are as many as 6562 reservoirs in Guangdong. The TSS retrieval results also show specific heterogeneity, that is, the spatial distribution characteristics of high upstream and low downstream, where the average concentration of TSS at 31.54 mg/L in the upstream is 2.5 times that of the downstream at 12.55 mg/L.

Generally, there were strong seasonal differences in the TSS concentration and TSS were susceptible to rainfall. When the rainfall intensity is small during the dry season, the concentration of TSS in water from upstream mankind activity is greater than the resuspension rate caused by rainfall, meaning that the TSS concentration is negatively correlated with rainfall. Meanwhile, in the flood season, since the rainfall intensity is greater, the settling rate of TSS in water is less than the resuspension rate caused by rainfall, meaning that the TSS concentration increases with the rainfall intensity. Besides, man-made interventions such as regular felling of eucalyptus and fruit tree planting also had a certain impact on TSS. 

As for the Markov dynamic evaluation of the TSS concentration, the water quality improvement effect of the upstream Shijiao Town water area is the most obvious, especially in 2018, the improvement in the water quality level crossed three levels and the TSS concentration was the lowest, which indicate the effectiveness of implementing the river chief system of China starting in September of 2017. The reasons for the TSS concentration decrease are due to the reduction in sediment mining activities and the improvement in the surrounding ecological environment, which also show that the river chief system has made great contributions to water quality monitoring and governance.

## Figures and Tables

**Table 1:** Summary of total suspended solids (TSS) concentration (N = 49) from the synchronous in situ spectra collected at different sites during several in situ campaigns.

Location	Date	Samples			TSS Concentration (mg/L)			
		Total	Calibration	Validation	Max	Min	Mean	Standard Deviation
Hedi Reservoir	August 2015	10	21	8	50	5	11.65	1.52
	October 2015	19						
Poyang Lake	June 2017	20	13	7	66	4	28.48	3.64
All	-	49	34	15	66	4	18.52	2.09

**Table 2:** Summary of the satellite remote sensing data used in this study, which includes a ground-synchronized remote sensing image on 16 October 2015.

Season	Type of Data	Image Date		Track Number
Dry season	Landsat8 OLI	14 Novmeber 2014	21 December 2016	Path: 124 Row: 45
		1 January 2015	8 December 2017	
		17 January 2015	24 December 2017	
		3 November 2016	6 January 2017	
		5 December 2016	22 January 2017	
	Sentinel-2	19 December 2018		49QDE
Flood period	Landsat8 OLI	9 July 2014	30 July 2016	Path: 124 Row: 45
		11 September 2014	16 September 2016	
		27 September 2014	14 May 2017	
		13 October 2014	30 May 2017	
		12 July 2015	18 August 2017	
		14 September 2015	3 September 2017	
		30 September 2015	18 June 2018	
		16 October 2015	6 September 2018	
		11 May 2016	8 October 2018	

**Table 3:** The relationship between the progress degree (pd(S)) and the TSS level transfer progress is expressed by using the variation in the TSS level (ΔL). Among them, pd(S) is the degree of progress calculated by the Markov model, which quantitatively describes the degree of change in the conversion between any two TSS concentration levels to indirectly reflect the water quality status of the water body; ΔL is the difference in the number of changes in the two TSS levels during the change in the TSS level status. Additionally, the division between TSS concentration levels is based on Chinese standards, mainly including 4 TSS levels, followed by I: 0 ≤ TSS < 20 (mg/L), II: 20 ≤ TSS < 30 (mg/L), III: 30 ≤ TSS < 50 (mg/L) and IV: TSS ≥ 50 (mg/L).

Progress Degree pd(S)	A Variation in TSS Level ΔL	TSS Level Transfer Process	Annotation
pd(s)∈(−16,−9]	−3	I→IV	TSS level has been reduced by 3 levels (from I to IV), water quality has deteriorated
pd(s)∈(−9,−4]	−2	II→IV, I→III	TSS level has been reduced by 2 levels (from II to IV or I to III), water quality has deteriorated
pd(s)∈(−4,−1]	−1	I→II, II→III, III→IV	TSS level has been reduced by 1 level (from I to II or II to III or III to IV), water quality has deteriorated
pd(s)∈(−1,1)	0	No change	TSS level has not changed, water quality remains stable
pd(s)∈[1,4)	1	IV→III, III→II, II→I	TSS level has been increased by 1 level (from IV to III or III to II or II to I), water quality has improved
pd(s)∈[4,9)	2	IV→II, III→I	TSS level has been increased by 2 levels (from IV to II or III to I), water quality has improved
pd(s)∈[9,16)	3	IV→I	TSS level has been increased by 3 levels (from IV to I), water quality has improved

**Table 4:** Review of previous TSS retrieval models and the comparison of the validation accuracy of several TSS retrieval models.

From		Study Area	Data	Model	Validation		
					N	RMSE (mg/L)	MRE (%)
Santiago Yepez et al. (2018)		Orinoco River	OLI Bands 5	$\mathrm{TSS}=1.35512b_{5}-2.9385$	15	10.79	47.28
Wang et al. (2009)		Yangtze River	ETM Bands 4	$\mathrm{TSS}=3.18236\ln(b_{4})-1.4006$	—		
Christopher Wackerman et al. (2017)		Mekong Delta	OLI Bands 2,4	TSS=e−2.9030b2b4−1.52	15	15.94	31.28
Muhammad Fauzi et al. (2016)		Wadaslintang Reservoir	OLI Bands 3,4	TSS=255.78b4b3−166.89	15	8.78	27.36
Wang et al. (2017)		Pearl River estuary	OLI Bands 4,5	$\begin{array}{l} \frac{{\log(b}_{5})}{{\log(b}_{4})}=-0.3575\log^{2}(\mathrm{TSS})\;+\;1.1135\log(\mathrm{TSS})+0.7162 \end{array}$	13	20.57	63.54
Hou et al. (2018)		Jiaozhou Bay	ETM Bands 2,3,4	TSS=9366.2[b4(1b2−1b3)]2−3878.6×b4(1b2−1b3)+407.94	—		
Zhang et al. (2015)		Xin’anjiang Reservoir	OLI Bands 2,3,8	$\begin{array}{l} \mathrm{TSS}=-191.02b_{4}+36.863b_{3}+172.66b_{8}+4.57 \end{array}$	15	12.85	34.16
This study	Band ratio model	Hedi Reservoir	OLI Bands 3,4	$\begin{array}{l} \mathrm{TSS}=172.191\ln^{2}(b_{3}/b_{4})-190.809\ln(b_{3}/b_{4})+61.6 \end{array}$	15	6.39	19.62
	Three-band model		OLI Bands 2,3,4	$\begin{array}{l} \mathrm{TSS}=-16.517\times \ln[[{\left(b_{4}\right)}^{-1}-{\left(b_{3}\right)}^{-1}]b_{2}]-8.363 \end{array}$	15	6.24	18.02

**Table 5:** Taking Shijiao Town of Hedi Reservoir as the research area, Markov evaluation was performed on the water quality using the pixel-based TSS and regional-averaged TSS data.

Years	Progress Degree pd(S)	
	Pixel-Based TSS	Region-Averaged TSS
2014	$pd\;(S_{2014})=2.493$	$pd^{’}(S_{2014})=0$
2015	$pd\;(S_{2015})=0.194$	$pd^{’}(S_{2015})=-3.333$
2016	$pd\;(S_{2016})=0.819$	$pd^{’}(S_{2016})=-2.667$
2017	$pd\;(S_{2017})=2.042$	$pd^{’}(S_{2017})=0.899$
2018	$pd\;(S_{2018})=10.232$	$pd^{’}(S_{2018})=0.667$
